# Primary Intraparenchymal Solitary Malignant Melanoma of the Brain Without Dural Attachment: A Rare Case With Local Recurrence

**DOI:** 10.7759/cureus.116631

**Published:** 2026-09-21

**Authors:** Muhammad Jamil Sarwar, Muhammad Waqas Umer, Abdullah Aslam, Haseeb Mehmood Qadri, Muhammad Hannan Tayyab, Muhammad Usman Malik, Asif Bashir

**Affiliations:** 1 Neurological Surgery, Punjab Institute of Neurosciences, Lahore, PAK; 2 Medicine, Aga Khan University Medical College, Karachi, PAK; 3 General Surgery, Lahore General Hospital, Lahore, PAK; 4 Neurological Surgery, Mayo Hospital, Lahore, PAK

**Keywords:** brain neoplasms, central nervous system neoplasms, local neoplasm recurrence, melanoma, primary neoplasms

## Abstract

Malignant melanoma is a neoplasm arising from the malignant transformation of melanocytes. These cells are derived from the neural crest and are found predominantly in the skin, but also in mucous membranes, the uveal tract of the eye, the leptomeninges, and other sites. A 39-year-old previously healthy male presented with a holocranial headache for two months and a single episode of a focal seizure involving his left arm. Neurological exam revealed left-sided pronator drift and gross papilledema. Contrast-enhanced magnetic resonance imaging (CE-MRI) of the brain revealed a large (4.8 x 3.9 cm) heterogeneously enhancing lesion in the right parieto-occipital region with marked perilesional edema, with the provisional diagnosis of a high-grade lesion. Complete metastatic work-up, including computerized tomography (CT) of the chest and abdomen and positron emission tomography (PET), revealed no primary lesion; hence, the lesion was planned for excision. Gross total excision (GTE) was achieved, and histopathological examination (HPE) of the resected specimen confirmed malignant melanoma. Recurrence was noted 11 months later with an even bigger lesion (6.0 x 5.0 cm) on CE-MRI, and the patient succumbed to death 45 days later under the care of oncology for adjuvant chemotherapy. Primary intracerebral malignant melanomas are extremely rare tumours of the CNS. Despite surgical and chemotherapeutic treatment options, these tumours are a clinical ordeal due to their aggressive behaviour and explosive growth rate. The primary existence of melanoma intracerebrally is an oncologic enigma and needs to be included in the differential diagnosis of primary brain lesions.

## Introduction

Malignant melanoma is most notorious for its cutaneous origin and its propensity to metastasize to the brain, where it ranks among the most common intracranial tumors in adults [1]. Far less recognized, however, is the ability of melanocytes to give rise to primary neoplasms within the central nervous system (CNS). These cells, embryologically derived from the neural crest, are normally found within the leptomeninges, and it is from this nidus that primary intracranial malignant melanomas (PIMMs) are believed to originate [2].

PIMMs are exceptionally rare, accounting for approximately 0.07% of all brain tumors [1]. Their clinical presentation, often characterized by headaches and focal deficits resulting from mass effect, is nonspecific and offers little to differentiate them from more common pathologies [2]. The diagnostic challenge is substantial; a solitary brain lesion found to be melanoma must first and foremost be assumed to be a metastasis until proven otherwise. This mandates a rigorous and exhaustive search for an occult primary tumor in the skin, mucosa, eyes, or other viscera, typically involving whole-body imaging and specialist consultations [1].

The existing literature, largely composed of single case reports and small series, has begun to clarify the behavior of these rare tumors. A systematic review by Arai et al., consolidating cases reported since 1992, emphasizes that gross total resection is the single most significant factor associated with prolonged survival [1]. Conversely, the presence of leptomeningeal enhancement on preoperative imaging portends a particularly grim prognosis.

Most documented cases of PIMM are found in relation to the meninges, such as the pineal region or the skull base [2]. A purely intraparenchymal lesion, particularly one without any connection to the dura or leptomeninges, is an even more unusual presentation that deviates from the expected origin of these tumors. This anatomical ambiguity adds another layer of complexity to an already difficult diagnosis.

Here, we present a rare case of a primary solitary malignant melanoma located deep within the cerebral parenchyma, with no evidence of dural attachment. The tumor's local recurrence following initial resection offers a valuable contribution to the limited body of knowledge on the natural history of these lesions. By discussing this case within the context of the current literature, we aim to highlight the challenges in diagnosis, the critical importance of excluding metastatic disease, and the need for continued surveillance even after seemingly complete surgical resection.

## Case presentation

A 39-year-old previously healthy male presented with a two-month history of progressive holocranial headache. One week prior to presentation, he experienced a single episode of focal seizure involving his left arm without loss of consciousness. There was no history of fever, weight loss, or visual disturbances. On examination, his vitals were stable. A detailed neurological assessment revealed a left-sided pronator drift, with motor power graded at 4/5 in both the left upper and lower limbs; sensory examination was normal. Fundoscopy identified bilateral papilledema. Based on this presentation, the initial broad differential diagnoses considered were a primary brain tumor (such as glioma or meningioma), a solitary brain metastasis from an occult primary.

A contrast-enhanced magnetic resonance imaging (MRI) scan of the brain was performed, which demonstrated a solitary, large (approximately 4.8 x 3.9 cm) heterogeneously enhancing mass located in the right parieto-occipital lobe. The lesion had irregular margins and was associated with marked perilesional edema and significant mass effect, causing midline shift (Figure 1). No dural tail sign was present. No other intracranial lesions were observed. Following this imaging, the differential diagnoses were narrowed to a high-grade glioma, a solitary metastasis, or a primary central nervous system lymphoma.

**Figure 1:**
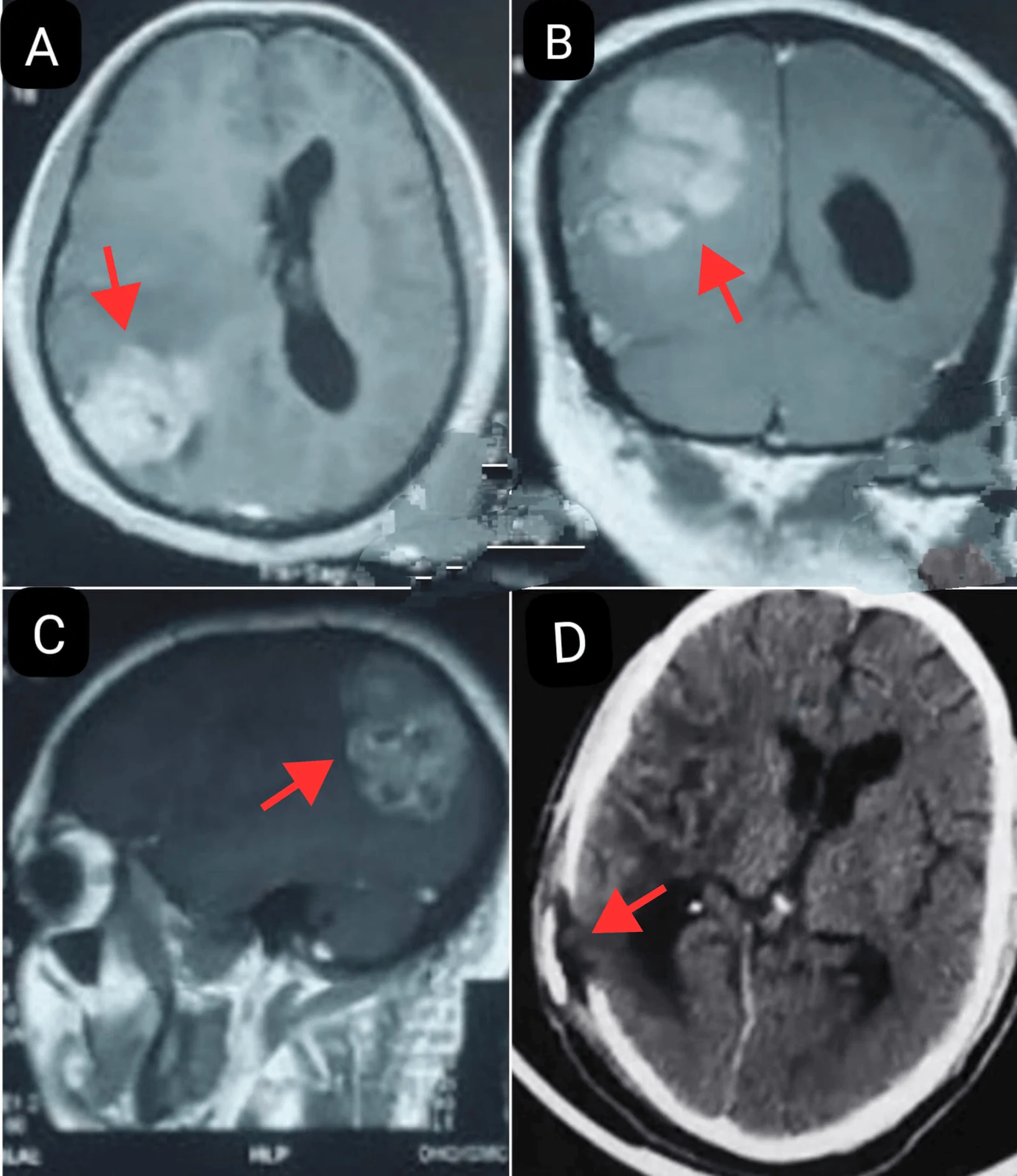
Pre-operative contrast-enhanced magnetic resonance imaging brain at initial presentation showing heterogeneously enhancing parieto-occipital lesion (red arrows) in (A) axial, (B) coronal and (C) sagittal sections. (D) Post-operative computerized tomography scan showing excision of the lesion with structural changes.

A complete metastatic workup, including contrast-enhanced CT scans of the chest and abdomen, was performed to identify a primary malignancy. These investigations were unremarkable. Laboratory investigations were all within physiological limits (Table 1).

**Table 1:** Summary of Laboratory Investigations at Presentation. WBC: white blood cell count; RBC: red blood cell count; HGB: hemoglobin; ESR: erythrocyte sedimentation rate; AST: aspartate aminotransferase; ALT: alanine aminotransferase; ALP: alkaline phosphatase; PT: prothrombin time; INR: international normalized ratio; APTT: activated partial thromboplastin time.

Tests	Result	Unit	Reference Range
WBC	9.8	×10³/µL	4.0 – 11.0
RBC	4.6	×10⁶/µL	3.8 – 5.2
HGB	14.5	g/dL	11 – 15.5
ESR	12	mm/hr	0 – 15
AST	25	U/L	8 – 33
ALT	49	U/L	7 – 56
ALP	119	U/L	42 – 306
Total Bilirubin	0.9	mg/dL	0.2 – 1.1
Serum Urea	36	mg/dL	15 – 40
Serum Creatinine	1.0	mg/dL	0.4 – 1.3
Sodium	139	mmol/L	135 – 154
Potassium	4.9	mmol/L	3.5 – 5.4
Chloride (Cl)	101	mmol/L	98 – 107
Glucose (Random)	186	mg/dL	70 – 199
PT	11	sec	11 – 14
INR	1.0	Unitless	1.0 – 1.3
APTT	26	sec	26 – 32

Given the solitary nature of the lesion, its significant mass effect, and the diagnostic uncertainty, a decision for surgical intervention was made. The patient underwent a right parieto-occipital craniotomy for gross-total resection of the tumour microsurgically. This was determined by the absence of macroscopically visible residual tumour on direct microscopic inspection of the resection bed by the operating surgeon at the conclusion of the procedure. Intraoperative frozen section and post-operative contrast-enhanced magnetic resonance imaging is not an institutional protocol due to profound resource limitations and affordability issues for the patient; microscopic residual disease could therefore not be formally excluded. Intraoperatively, the tumour was found to be firm, highly vascular, and intensely black in colour. It was not adherent to the overlying dura. A clear plane of cleavage from the underlying brain parenchyma was established. The postoperative recovery was uneventful, with no new neurological deficits.

Histopathological examination of the resected specimen revealed a malignant neoplasm composed of sheets and nests of epithelioid cells with abundant cytoplasm (Figure 2). The nuclei were markedly pleomorphic, hyperchromatic, and exhibited prominent eosinophilic nucleoli. Scattered mitotic figures were readily identifiable. Critically, the tumour cells contained abundant coarse, dark brown intracytoplasmic pigment, which was confirmed to be melanin by a positive Fontana-Masson stain. Immunohistochemical staining was strongly positive for S-100, HMB-45, and Melan-A, confirming the melanocytic origin of the neoplasm. Based on these morphological and immunohistochemical features, a diagnosis of malignant melanoma was made.

**Figure 2:**
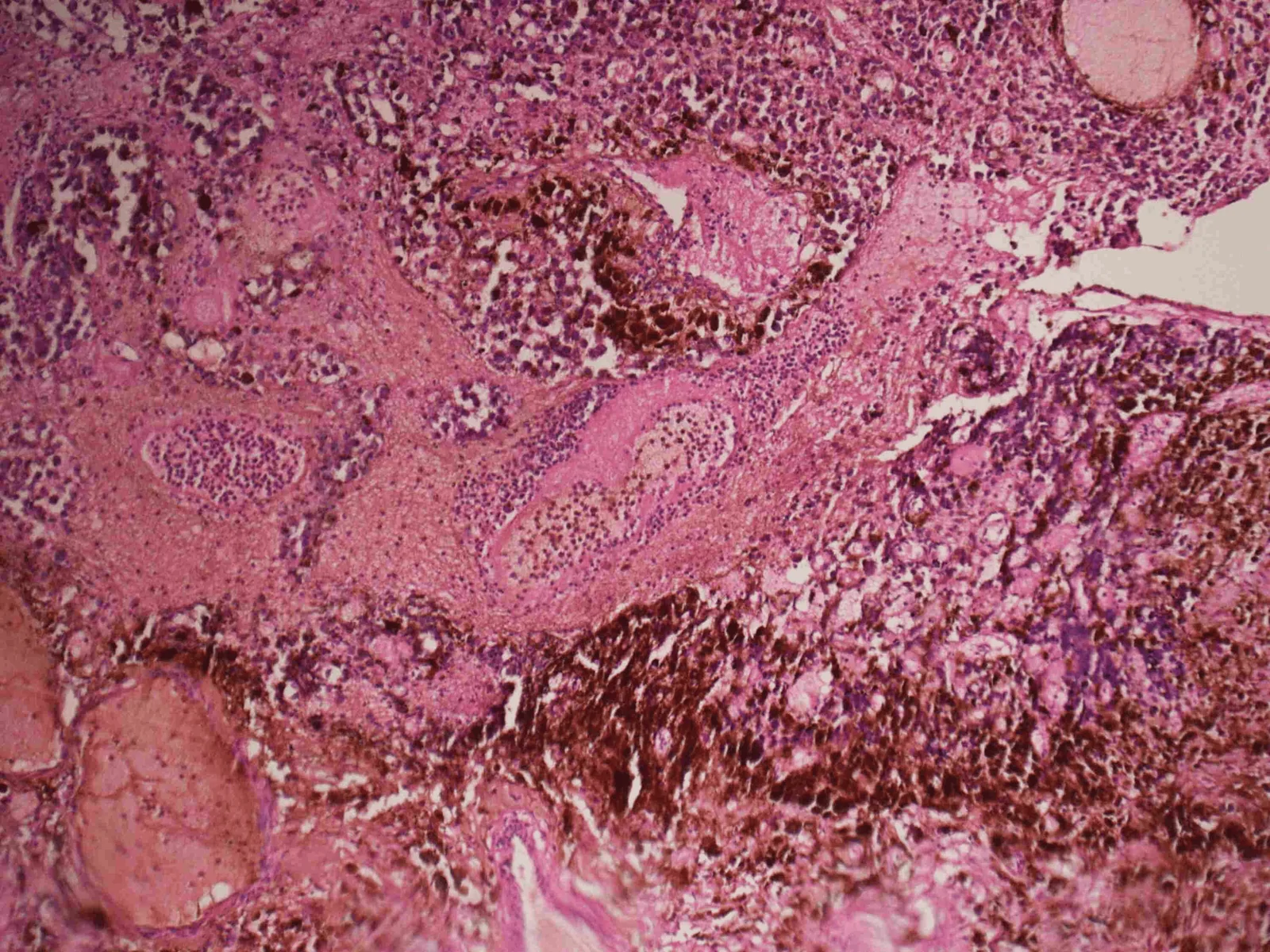
Light microscopy with hematoxylin and eosin stains under 10x power reveals pleomorphic malignant melanocytic cells with brown pigment. The cells are arranged around thick-walled blood vessels.

The patient was discharged in stable condition. At a 7-day follow-up visit, the surgical wound was healing well. As our centre lacks a dedicated medical oncology service, he was referred to the INMOL Hospital, Lahore, for further management. At the oncology centre, a whole-body 18F-fluorodeoxyglucose positron emission tomography-computed tomography (18F-FDG PET-CT) scan was performed for comprehensive staging before initiating adjuvant therapy. The scan showed no FDG-avid primary lesion in the skin, mucosa, or retina, and no evidence of regional lymph node or distant metastatic disease elsewhere in the body (Figure 3). The patient was subsequently referred for consideration of radiotherapy and chemotherapy. However, he was unfortunately lost to follow-up from our neurosurgical service after this referral.

**Figure 3:**
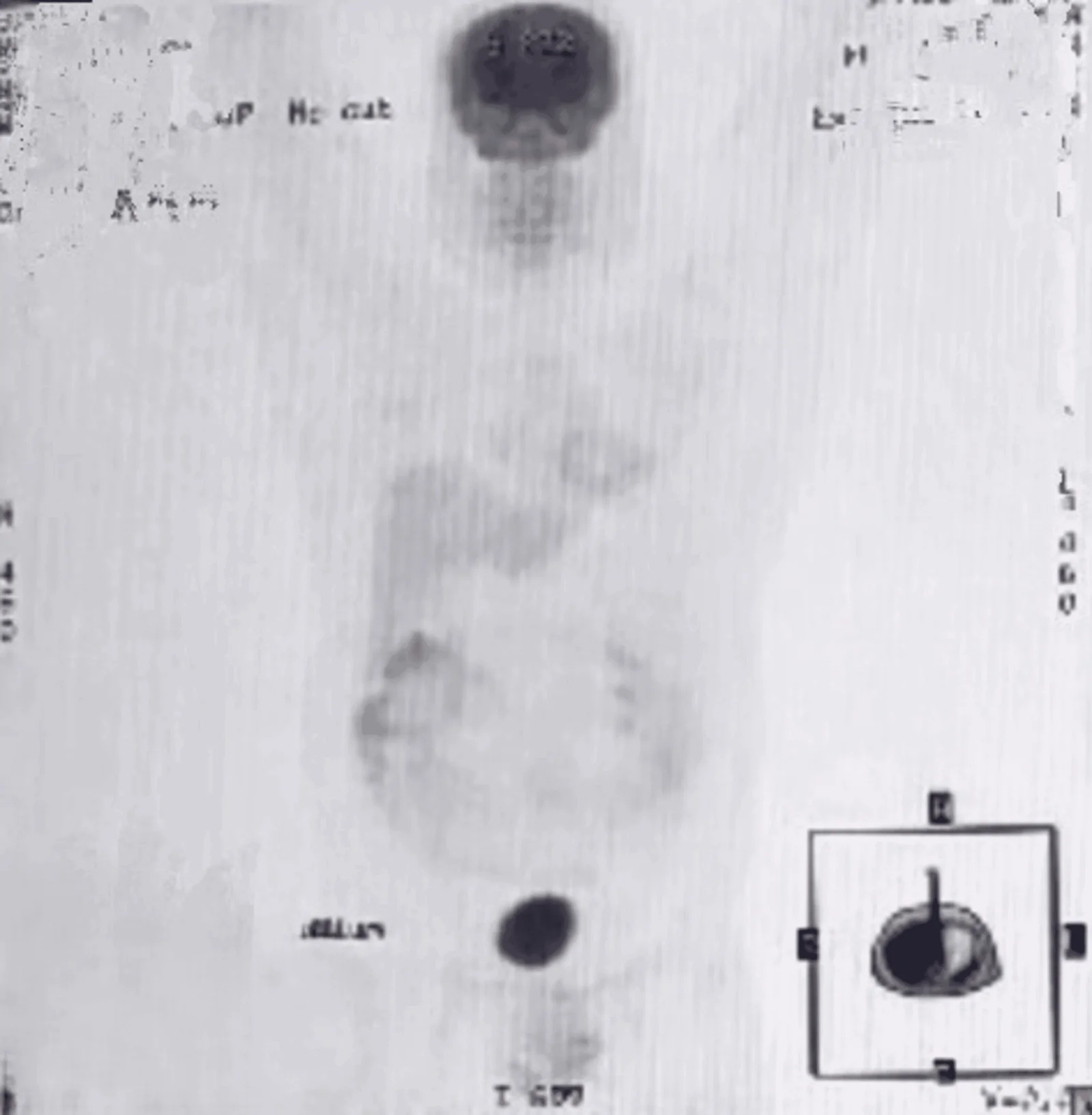
18F-fluorodeoxyglucose positron emission tomography-computed tomography (18F-FDG PET-CT) scan without any evidence of regional lymph node or distant metastatic disease elsewhere in the body.

Eleven months following his initial surgery, the patient re-presented with progressive left-sided hemiparesis and altered sensorium. A repeat MRI brain revealed a large recurrent mass lesion, measuring approximately 6.0 x 5.0 cm, at the previous operative site, causing significant mass effect. He underwent a second craniotomy for maximal safe resection of the recurrent tumour (Figure 4). His postoperative recovery was uncomplicated, and he remained neurologically stable and symptom-free for a period of four months. Thereafter, he developed generalised lymphadenopathy and was managed by the medical oncology team. He succumbed to death 45 days later under care.

**Figure 4:**
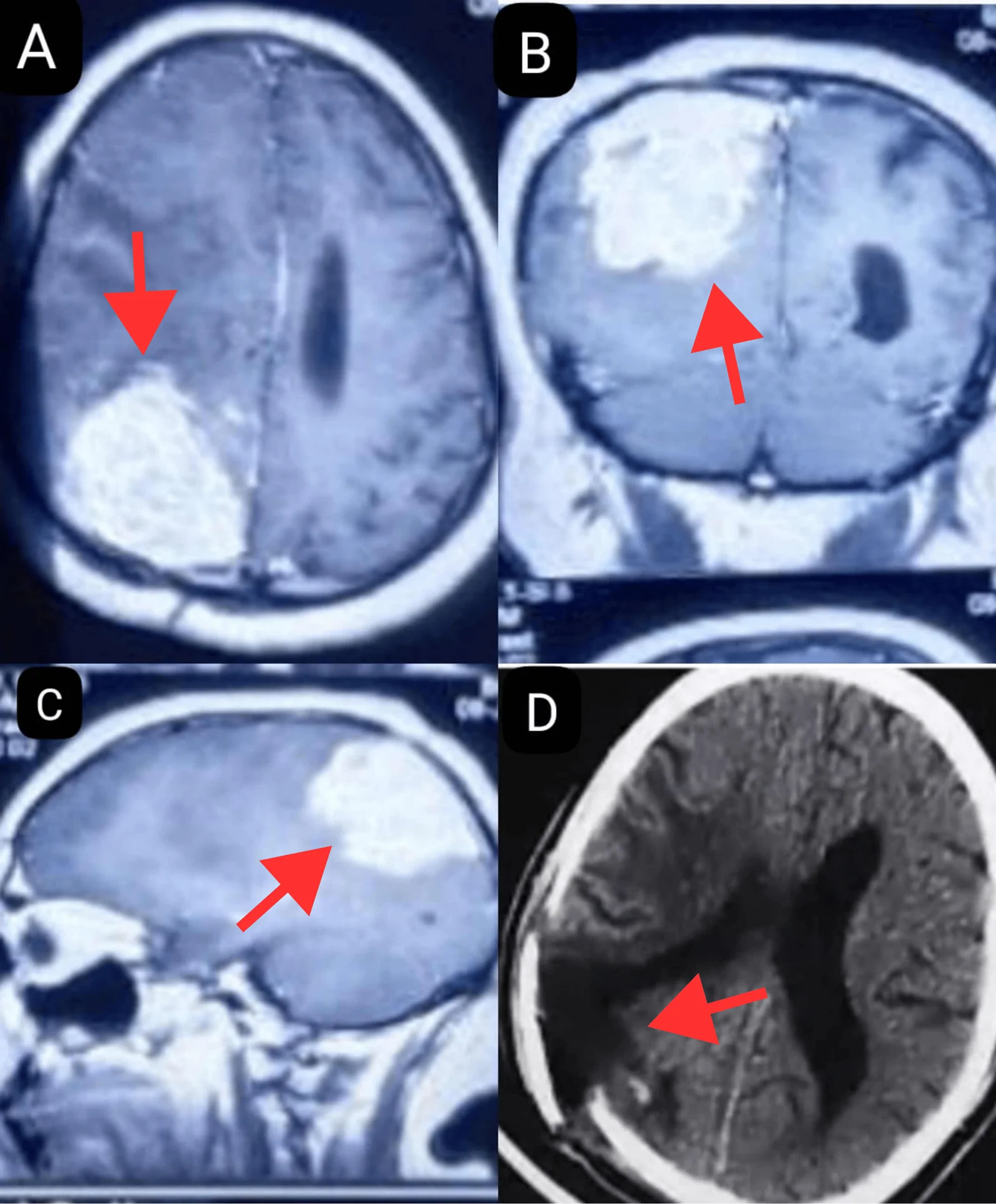
Pre-operative contrast-enhanced magnetic resonance imaging brain at recurrent presentation showing heterogeneously enhancing parieto-occipital lesion (red arrows) in (A) axial, (B) coronal and (C) sagittal sections. (D) Post-operative computerized tomography scan showing excision of the lesion with structural changes.

Normal peripheral clinical examination, the solitary nature of the lesion on neuro-imaging, negative whole-body imaging (CT and PET-CT) for a primary source and recurrence of the same lesion in the primary location were the reasons we labelled it as a case of primary right parieto-occipital malignant melanoma.

Informed written consent for case management and publication of this case (including images) was obtained from the patient.

## Discussion

Metastatic melanomas have a high propensity to spread into the CNS. They are usually characterised by a rapid, poor clinical course due to systemic metastasis, multiple intracerebral lesions, and common development in older patients [3]. The primary tumour is usually found in 90% of such cases, while it remains unknown in the other 10% [4]. Cerebral melanomas with an unknown primary or primary intracranial melanomas are therefore rare and account for nearly 1% of all melanomas [5]. Our case shows the presence of a single solitary mass in the CNS of a relatively young male patient. The lesion was histologically confirmed to be a melanoma, with no primary extracranial tumours.

Primary melanomas in the CNS may arise from melanocytes present in the leptomeninges, and diagnosis of such cases is complex and requires the exclusion of an extracranial primary tumour [2]. This is further complicated when there are possibilities of a spontaneously regressed cutaneous primary or an occult mucosal lesion. Our case strengthens the contrasting presentation of primary and secondary melanomas reported in the literature as the patient was a relatively young male with the common presenting complaints of headache, hemiparesis, and seizures [3]. Moreover, his solitary intracranial lesion tested strongly positive for S-100, HMB-45, and Melan-A, which is a repeated pattern in reported cases [6]. Unlike most cases, however, our patient’s lesion had no dural attachment.

Our patient also presented with recurrence of the primary tumour at 11 months post-initial surgery. This early local recurrence can be attributed to several factors such as microscopic residual disease or aggressive tumour biology. However, regardless of the mechanism, early recurrence has a major impact on the prognosis of such cases, as further surgery or adjuvant therapy may become necessary for maximum survival. The recurrence in our patient conforms to the pattern evident in the literature, as recurrence is often reported within 18 months of initial surgery [3]. This supports the possibility of a similar aggressive CNS phenotype being reported in multiple instances.

The diagnosis of a primary cerebral melanoma requires an exhaustive systemic workup as it is commonly a diagnosis of exclusion [2]. Physicians should examine probable sites of a primary melanoma that include skin, mucous membranes, GI tract, and eyes [3]. Moreover, systemic imaging such as contrast-enhanced CT scans and 18F-FDG PET scans is required to rule out the presence of a primary lesion [1]. However, these modalities are still limited in their ability to definitively rule out regressed or occult primaries. This makes them evaluative rather than confirmatory. Such limitations must be taken into account, especially when dealing with rare and complicated cases.

Several treatment options for primary intracranial melanomas have been recommended based on specific characteristics such as size and location of the lesion [7]. These include resections or biopsy, along with adjuvant therapy such as stereotactic radiosurgery, chemotherapy, or immunotherapy [8]. Recent literature has also recommended the use of immunotherapy and targeted therapy against malignant melanomas after molecular analysis, especially against the commonly encountered BRAFV600E mutation [1,9]. Maximal safe resection with adjuvant radiation and chemotherapy is considered a first-line treatment option for both primary intracranial as well as metastatic intracranial melanomas. Due to the complexity of the cases and the vast range of treatment options available, a multidisciplinary approach is required for optimal individualised therapy.

Solitary intracerebral melanomas are rare occurrences that present with clinical uncertainty and unique diagnostic as well as treatment challenges. A review of the literature on primary intracranial melanomas from Pakistan has been summarised in Table 2. Both our case and the relevant reported literature emphasise the importance of long-term surveillance because of high recurrence rates and the complex nature of the condition. In this way, our case strengthens the currently available literature on the topic by adding to our understanding of melanomas of the CNS, their patterns, behaviours, and prognosis.

**Table 2:** Summary of Literature Review on Primary Intracranial Melanoma Cases from Pakistan GTR: gross total resection; MSR: maximal safe resection

Study By	Age	Gender	Site and Size of CNS Melanoma	Treatment	Outcome
Shah et al. [4]	28 years	Female	Left Temporal Lobe	GTR	Recurrence-free for 4 years
Saleem et al. [9]	62 years	Male	Left Temporal Lobe 37 × 41 × 32 mm	GTR	Metastasis to Spine, Death in 10 months
Hassan et al. [10]	36 years	Male	Posterior Fossa 42 × 35 × 20 mm	MSR	Unremarkable recovery
Khan et al. [11]	39 years	Female	Left Insular Region 41 x 42 x 38 mm	Biopsy	Cerebral edema, Death in 5 months

## Conclusions

Primary intracerebral malignant melanomas are rare tumors of the CNS, with complex biological and clinical pictures. Despite the large number of treatment options, these tumors remain difficult to treat because of their associated biological and clinical uncertainty. The use of radiological imaging, molecular analysis, and histopathological examination is essential for confirmatory diagnosis and for desirable treatment outcomes. Therefore, this case highlights the need for comprehensive investigations and multidisciplinary decision-making when handling rare pathologies, especially of the CNS.
